# Comparison of conventional and wide field direct ophthalmoscopy on medical students’ self-confidence for fundus examination: a 1-year follow-up

**DOI:** 10.1186/s12909-021-02942-y

**Published:** 2021-09-26

**Authors:** Gabriel Ayub, Rafael Boava Souza, Andrelisa Marina de Albuquerque, José Paulo Cabral de Vasconcellos

**Affiliations:** Department of Ophthalmology, Faculty of Medical Sciences, University of Campinas, 251 Vital Brazil St, SP 13083-888 Campinas, Brazil

**Keywords:** Direct ophthalmoscopy, Fundus examination, Self-confidence, Medical student education, Questionnaire validation

## Abstract

**Background:**

Fundus examination is an easy, quick and effective way to diagnose sight- and life-threatening diseases. However, medical students and physicians report lack of proficiency and self-confidence in perform fundoscopy. The aim of this study was to compare students’ self-confidence in fundus examination, using two different direct ophthalmoscopes, 1 month and 1 year after practical training.

**Methods:**

In this prospective cohort, medical students (MS) of the same class were divided in small groups for PanOptic (PO) or conventional (CO) direct ophthalmoscope training. The intervention group encompassed MS of the 4th -year (class of 2019), and the control group encompassed MS of year behind (class of 2020). A questionnaire to measure self-confidence in fundoscopy technique assessing optic nerve, cup-to-disc ratio and macula was translated and validated to Portuguese, and applied 1-month and 1-year after practical training.

**Results:**

One-hundred and sixty-seven MS were enrolled (35 PO group, 38 CO group, and 94 control group). PO group had a significantly higher overall self-confidence comparing either control or CO groups, respectively (3.57 ± 0.65 vs. 2.97 ± 1.03 vs. 2.46 ± 0.87, *p* < 0.01) as well as in evaluate cup-to-disc ratio (3.09 ± 0.75 vs. 2.32 ± 0.87 vs. 1.46 ± 0.81, *p* < 0.01), optic disc margins (3.26 ± 0.85 vs. 2.71 ± 0.96 vs. 2.01 ± 0.97, *p* < 0.01) and macula (3.43 ± 1.12 vs. 2.89 ± 1.08 vs. 2.02 ± 0.89, *p* < 0.01) 1-month after practical training. One-year after intervention, CO group showed a significantly higher score compared to PO group in overall self-confidence (3.31 ± 0.69 vs. 3.18 ± 0.73, *p* = 0.03) and in optic disc margins assessing (3.16 ± 0.85 vs. 2.95 ± 0.78, *p* = 0.03), but not significant in the evaluation of cup-to-disc ratio (2.78 ± 0.97 vs. 2.68 ± 0.94, *p* = 0.08), and macula (3.34 ± 0.79 vs. 3.27 ± 0.98, *p* = 0.07).

**Conclusions:**

Students were more confident in use PO as an instrument to perform direct ophthalmoscopy immediately after practical training, but confidence level of CO was higher compared to PO one year after practical training. These findings would help medical schools decide which ophthalmoscope to choose to teach fundus examination.

**Supplementary Information:**

The online version contains supplementary material available at 10.1186/s12909-021-02942-y.

## Background

Reversible blindness has been increasing worldwide. In 2020 was estimated that 237 million people had moderate to severe vision impairment, while 38 million were blind [1]. The leading causes were refractive errors, cataract, glaucoma, age-related macular degeneration and diabetic retinopathy [1]. A strategy to provide eye care and early diagnosis would reduce the burden of reversible blindness worldwide.

Fundus examination is still an easy, quick and effective way to diagnose sight- and life-threatening diseases in emergency [2] and primary-care environments [3–6]. A plenty of devices are available for fundus examination: indirect ophthalmoscopy, retinography, direct ophthalmoscopy, and more recently, smartphone-based ophthalmoscopy (SF) [7]. Indirect ophthalmoscopy demands more training time and additional instruments to perform fundus examination; although the emergence of SF, it remains a high-cost examination [7]. The International Council of Ophthalmology [8, 9] and the Association of University Professors of Ophthalmology [10] recommend teach fundus examination to medical students (MS) for a proficiency in handling the device, direct ophthalmoscope, and identify normal and abnormal fundus and optic nerve head. Despite recommendations, the art of fundoscopy has been forgotten [11] due to a crowded curriculum in medical schools, which reduced the time of ophthalmology rotation [12], fall of ownership of ophthalmoscopes [13] and no consensus on minimum ophthalmoscopy proficiency [14, 15], which results in a lack of knowledge and self-confidence in perform fundoscopy by general practitioners [5, 16–20].

Among direct ophthalmoscopes devices, wide field ophthalmoscope provides a larger field of view and an increased magnification compared to conventional ophthalmoscope. Besides, some studies have reported to be easier to use it in fundus examination [2, 21]. A major disadvantage of wide field ophthalmoscope, however, is its higher price compared to conventional devices.

Some studies have addressed the apprenticeship of the ophthalmoscopy exam during the MS graduation. They have observed an increase in MS’s knowledge and self-confidence soon after a practical training [18, 22], but both seems to decrease with time [19, 20, 23]. A longer follow-up would address the effectiveness of hands-on fundoscopy to recognize the most relevant structures in fundus examination. In addition, MS’ self-confidence in conducting fundus examination with different ophthalmoscopes, such as a wide-field, was not investigated one year after a practical training, according to our knowledge. The aim of our study was to evaluate students’ self confidence in two different ophthalmoscope devices 1 month and 1 year after practical training.

## Methods

This prospective cohort study was approved by the Research Ethics Committee of the University of Campinas and conducted in compliance with the Declaration of Helsinki. All procedures were fully explained and an informed consent was obtained from all participants.

Participants were recruited from July/2019 to January/2020 and a new one-year evaluation was done between October/2020 and November/2020.

### Inclusion and exclusion criteria

For contextualization, the undergraduate medicine course in Brazil takes 6 years. At University of Campinas the course is divided in two moments: second year (basic rounds) and fourth year (clinical rounds). The basic rounds consist of theoretical lectures, which exposes the anatomy, physiology and histology of the eye and the vision, in a total of 6 h. The clinical rounds also consist of theoretical lectures, focused on basic eye exam and pathologies, and a basic practical training on physical examination of the eye on slit lamp, visual acuity check, fundus examination and surgical observation, encompassing all sub-specialties of the ophthalmology (retina, cornea, glaucoma, ocular plastics, strabismus, neuro-ophthalmology), in total of 36 h. Students are divided in subgroups of 6–7 persons for this practical training.

For classes in fundus examination, the intervention group included all MS of the 4th -year (class of 2019 at Faculty of Medical Sciences, University of Campinas). For the control group, all MS of the 4th -year (class of 2020 at Faculty of Medical Sciences, University of Campinas) were invited to participate. MS with either previous experience on managing an ophthalmoscope or students that missed the fundus examination classes as well as those who did not accept participate were excluded.

The ophthalmoscopes we used to teach fundus examination were a conventional ophthalmoscope (*Coaxial, Welch Allyn, Skaneateles Falls, NY, USA)* and a wide field ophthalmoscope (*PanOptic, Welch Allyn, Skaneateles Falls, NY, USA)*. Briefly, PanOptic is a wide field ophthalmoscope, which provides a 5x larger field of view than conventional Coaxial ophthalmoscope (25° versus 5°) and a 26 % increase in magnification. Also, PanOptic can be used in non-mydriatic patients.

### Questionnaire translation and cross-cultural validation

We included residents from first- to fourth-year for the questionnaire translation and validation. The questionnaire developed by Haque et al. [24] was used as basis. This questionnaire was design and validated to rank student’s confidence in identify eye fundus structures as disc margins, cup-to-disc ratio, macula and the overall confidence on using the ophthalmoscope, based on a Likert scale ranked from 1 (low confidence) to 5 (extreme confidence).

The process for translation and cross-cultural validation was previously described by several authors [25–28]. First, the basis questionnaire was translated from English to Portuguese by 2 native Portuguese speakers. The translated questionnaire was then evaluated by a committee composed by 2 ophthalmologists and 2 MS to ensure an adequate translation and cross-cultural validation. Later, 2 native English speakers back translated the questionnaire from Portuguese to English, which was then re-checked by the committee.

Finally, we applied the Portuguese version of the questionnaire in a group of first- to fourth-year ophthalmology residents. Internal consistency, to evaluate the inter-correlation of the questionnaire items, was calculated using coefficient alpha (Cronbach’s alpha - α), which varies from 0 to 1, and a value > 0.7 is considered adequate [27]. One week later, the questionnaire was re-applied on the same group to verify test-retest reliability, calculated by Pearson correlation (R), and inter-rater reliability, calculated by Cohen’s Kappa (κ), which varies from 0 to 1, and is classified as: <0.2, poor agreement; 0.2–0.4, weak agreement, 0.4–0.6, moderate agreement; 0.6–0.8, good agreement; >0.8 excellent agreement [26, 27].

### Training of the participants and evaluation

Intervention group of MS of the 4th -year/2019 were divided in 2 groups after fulfilled the inclusion criteria: one group was designated to use the Coaxial ophthalmoscope (CO) in all the interventions, while the other, to use the PanOptic ophthalmoscope (PO).

Our intervention in teaching eye fundus examination was composed of a 6 h of practical training applied in subgroups containing 6–7 students. Our interventions were done by the same instructors (G.A. and J.P.C.V). First, we did a 1-h training with an initial explanation of the fundoscopy technique. Then, we used an acrylic cube model that simulates a normal eye fundus provided by Welch Allyn as first practical training of the technique, which the students could evaluate the main structures of the eye fundus (optic disc, cup-to-disc ratio, macula, vessels and retina). Later, students trained the technique among them to visualize the same structures in a normal human eye. Next, we helped students to examine dilated patients to identify normal and pathological structures, where findings compatible with diabetic retinopathy (intraretinal hemorrhages, hard exsudates, retina and optic disc neovascularization), hypertensive retinopathy (arteriovenous nicking and narrowing, cotton-wool spots), age-related macular degeneration (macular drusen) and glaucoma (increased cup-to-disc ratio) were visualized. All patients were prior evaluated by the instructor.

We applied the questionnaire in the control group at the first lecture day of the class in 2020 for a baseline of the MS confidence before intervention. The control group had the same experience in fundus examination than the intervention group before starting the 4th -year.

To check the 1-year retention of the intervention, we re-applied the same questionnaire on the same individuals of the intervention group between October/2020 and November/2020. All students where on the 5th -year at the time. Because of Covid-19 restrictions, application was virtual.

### Statistical analysis

Internal consistency (Cronbach’s alpha - α), test-retest reliability (Person correlation - R) and inter-rater reliability (Cohen’s Kappa - κ) were calculated as previously described.

Descriptive data are shown in percentage and mean ± standard deviation. Normality was assessed by QQ-Plot and inspecting a histogram. Baseline values where compared with ANOVA (age) and qui-square (gender). To compare the scores within 1-month between the intervention groups and control group, Kruskall-Wallis test was done, followed by Mann-Whitney-U test to compare each pair of groups. For this first comparison, the null hypothesis was considered as there was no difference between the groups, while the alternative hypothesis was considered as there would be a difference for one device or another (two-tailed tests). Mann-Whitney-U test was also used to compare CO and PO groups 1-year score after intervention. Moreover, Wilcoxon rank sum test was used to compared the paired scores between 1-month and 1-year intervention. For these two comparisons, the null hypothesis was also considered as there was no difference between the groups 1 year later, while the alternative hypothesis was considered as there would be a difference for one device or another (two-tailed tests).

Analysis were done with Statistical Package for Social Sciences – SPSS (*IBM Corporation, Armon NY, USA, version 22.0*).

## Results

### Questionnaire validation

A total of 35 ophthalmology residents were included in the questionnaire validation process. The translated questionnaire had a strong internal consistency (α = 0.82), and a good agreement with strong correlation in the overall confidence on the fundoscopy technique question (*R* = 0.83, κ = 0.61, *p* < 0.01) and optic disc margins question (*R* = 0.74, κ = 0.64, *p* < 0.01). Cup-to-disc ratio (*R* = 0.78, κ = 0.51, *p* < 0.01) and macula (*R* = 0.80, κ = 0.53, *p* < 0.01) questions had a moderate agreement with strong correlation [Table 1].

**Table 1:** Validation tests of the translated questionnaire. Test-retest reliability showed a strong correlation, and inter-rater reliability showed a good agreement in the fundoscopy technique and optic disc margins questions, and a moderate agreement in the cup-to-disc ratio and macular evaluation question. Internal consistency was α = 0.82

	Pearson correlation (test-retest reliability)	Cohen’s kappa (inter-rater reliability)	*P*-value
Question 1: fundoscopy technique	0.83	0.61	**< 0.01**
Question 2: cup-to-disc ratio	0.78	0.51	**< 0.01**
Question 3: optic disc margins	0.74	0.64	**< 0.01**
Question 4: macula evaluation	0.80	0.53	**< 0.01**

### One-month assessment

A total of 167 MS were included: 94 in the control group, 38 in the CO group and 35 in the PO group; baseline characteristics are depicted in Table 2. Students’ self-confidence score in the fundoscopy technique question was 2.46 ± 0.87 vs. 2.97 ± 1.03 vs. 3.57 ± 0.65 (*p* < 0.01), cup-to-disc ratio score was 1.46 ± 0.81 vs. 2.32 ± 0.87 vs. 3.09 ± 0.75 (*p* < 0.01), optic disc margins score was 2.01 ± 0.97 vs. 2.71 ± 0.96 vs. 3.26 ± 0.85 (*p* < 0.01) and macula score 2.02 ± 0.89 vs. 2.89 ± 1.08 vs. 3.43 ± 1.12 (*p* < 0.01) comparing control group, CO group and PO group, respectively [Table 3] The PO group was superior to the control group in all the 4 question (*p* < 0.01) and to the CO group in the fundoscopy technique (*p* = 0.02) and cup-to-disc ratio (*p* = 0.01) questions, but not in the optic disc (*p* = 0.13) and macula evaluations (*p* = 0.22) questions [Table 4]. Also, CO group had a higher score in all the 4 questions (question 1, *p* = 0.01; question 2, *p* < 0.01; question 3, *p* = 0.01; and question 4, *p* < 0.01) compared to the control group.

**Table 2:** Baseline characteristics of the groups. Values are presented as mean ± standard deviation. *P*-value was calculated with *ANOVA (age) and ^†^qui-square (gender)

	Control	Conventional	PanOptic	*P*-value
n	94	38	35	-
Age (years)	23.36 ± 2.30	24.41 ± 5.19	23.44 ± 2.44	0.24*
Gender (Male/Female)	37/57	14/24	17/18	0.55^†^

**Table 3:** One-month score of the 3 groups in each question. Values are presented as mean ± standard deviation. The PanOptic group was superior compared to the other groups. Kruskal-Wallis test was used to compare the groups

	Control	Conventional	PanOptic	*P*-value
Overall self-confidence	2.46 ± 0.87	2.97 ± 1.03	3.57 ± 0.65	**< 0.01**
Cup-to-disc ratio	1.46 ± 0.81	2.32 ± 0.87	3.09 ± 0.75	**< 0.01**
Optic disc margins	2.01 ± 0.97	2.71 ± 0.96	3.26 ± 0.85	**< 0.01**
Macula	2.02 ± 0.89	2.89 ± 1.08	3.43 ± 1.12	**< 0.01**

**Table 4:** Comparison between 1-month and 1-year scores. Values are presented as mean ± standard deviation. Statistically significant values are in bold. ^†^Wilcoxon rank sum test was used to compare the scores of medical students that completed the questionnaire in both periods, and *Mann-Whitney-U test were used to compare the groups within the same period

		Conventional	PanOptic			*P*-value
Overall self-confidence						
1-month	2.97 ± 1.03			3.57 ± 0.65	0.02*	
1-year	3.31 ± 0.69			3.18 ± 0.73	0.03*	
***P*-value**	0.22^†^			**0.01**^†^		
Cup-to-disc ratio						
1-month	2.32 ± 0.87			3.09 ± 0.75	0.01*	
1-year	2.78 ± 0.97			2.68 ± 0.94	0.08*	
***P*-value**	**0.01**^†^			0.09^†^		
Optic disc margins						
1-month	2.71 ± 0.96			3.26 ± 0.85	0.13*	
1-year	3.16 ± 0.85			2.95 ± 0.78	0.03*	
***P*-value**	**0.01**^†^			0.56^†^		
Macula						
1-month	2.89 ± 1.08			3.43 ± 1.12	0.22*	
1-year	3.34 ± 0.79			3.27 ± 0.98	0.07*	
***P*-value**	**< 0.01**^†^			0.64^†^		

### One-year assessment

One year after the intervention, the groups were re-evaluated, and 32 (84.21 %) students of the CO and 22 (62.85 %) in the PO group answered the questionnaire. Students’ self-confidence score in the fundoscopy technique question was 3.31 ± 0.69 vs. 3.18 ± 0.73 (*p* = 0.03), cup-to-disc ratio score was 2.78 ± 0.97 vs. 2.68 ± 0.94 (*p* = 0.08), optic disc margins score was 3.16 ± 0.85 vs. 2.95 ± 0.78 (*p* = 0.03) and macula score was 3.34 ± 0.79 vs. 3.27 ± 0.98 (*p* = 0.07) comparing CO group and PO group, respectively  [Table 4].

Comparison between scores 1-month and 1-year scores were higher and statistically significant for cup-to-disc ratio (*p* = 0.01), optic disc margins (*p* = 0.01) and macula evaluations (*p* < 0.01) but not for overall confidence (*p* = 0.22) in the CO group. In the PO group, the overall confidence was lower and statistically significant (*p* = 0.01), this did not present for cup-to-disc ratio (*p* = 0.09), optic disc margins (*p* = 0.56) and macula (*p* = 0.64) [Table 4].

## Discussion

### Questionnaire translation and validation

Translation and cross-cultural validation allows comparison between results obtained in two different cultures and populations by the same instrument. The questionnaire of Haque et al. [24] was chosen due to assess main structures of the eye fundus for general practice with a short instrument. Compared to them, who enrolled 13 s-year MS and 17 residents in a cross-sectional design, an overall confidence of 3.12, with the lowest score in cup-to-disc ratio, was found. Our study observed slightly lower value in CO and a higher value in PO group 1 month after training, and also reported the lowest confidence score in cup-to-disc ratio evaluation.

### The role of fundus examination in physical examination

One aspect that motivated our study was a perception in our institution of unfamiliarity of clerks, residents and general practitioners with the ophthalmoscope handling, identification of normal and pathological structures and self-confidence in perform the exam. This was investigated by Wu et al. [16], who measured the confidence of third- and fourth-year MS, first-and second-fourth-year internal medicine residents and general internists in perform 14 items of the physical examination with a 5-point Likert scale. While measure blood pressure achieved the highest score (4.4 ± 0.7 vs. 4.8 ± 0.5 vs. 4.4 ± 0.7 vs. 4.7 ± 0.5 vs. 4.9 ± 0.2, respectively, overall = 4.7 ± 0.6), non-dilated fundoscopy had the lowest score (2.3 ± 1.1, 2.8 ± 1.2, 2.3 ± 1.1, 2.2 ± 1.0, 3.2 ± 1.1, respectively, overall = 2.5 ± 1.1). Also, fundus examination had the third worst score among perceived utility, 3.7 ± 0.9, versus 4.9 ± 0.3 of measure blood pressure, the highest score, which reveals that both MS and graduated doctors have a lack of confidence and motivation to perform fundoscopy.

### Short-term evaluation

At short-term, practical training in fundoscopy appears to increased MS’ skills. Cordeiro et al. [18] validated a 8-question objective structured clinical examination questionnaire and assessed 29 MS skills in ophthalmoscopy before and after formal instruction. The authors measured a 23.7 % increase in MS’s skills (5.3379 ± 2.252 vs. 7.7069 ± 1.724, *p* < 0.001). Kelly et al. [22] compared direct ophthalmoscopy examination of human volunteers and human models simulators to fundus photograph in a randomized study that enrolled 119 first-year MS. Of a 48-item questionnaire, pre-test score was 60 %, while post-test score was 77 % for simulator and 85 % for photograph group (*p* < 0.001). In addition, 71 % of students preferred human volunteers to human models simulators, 77 % preferred photographs to human models simulators and 70 % preferred fundus photograph than use a direct ophthalmoscope. Our results show that both groups had a better performance one-month after the practical training, which reinforces what is observed in the literature.

### Long-term evaluation

Despite of an increase of MS’s skills after a practical training, these seem to decrease with time. Mackay an colleagues [23] reported the 1-year follow-up of the original study of Kelly et al. previously described [22], and revealed MS’s more accurate diagnosis and their preference for photograph when compared to CO. At 1-year retention study [23], out of 48 questions, students answered correctly 72 % with fundus photographs and 65 % with direct ophthalmoscopy (*p* = 0.004). This represented 5 fewer questions answered correctly at 1-year comparison in both groups (*p* < 0.001). A general discomfort (38 %), discouragement by preceptors (20 %) and insufficient time (15 %) were nominated as the main issues for not perform fundus exam. Also, in a 3-year follow-up study, Lippa and colleagues [19] evaluated a group of second-year MS longitudinally on third- and fourth-years after a 12-item training in eye screening skills. Of the 96 MS included in the first evaluation, 76 %±9 % felt comfortable to describe at least 1 characteristic of the visualized optic disc. One year later, the 96 MS had their fundus examination skills evaluated with a human model simulator: 32 % described some feature of the optic disc, but 22 % were unable to described correctly a fundus of a dilated eye and 20 % unable to complete the same in an undilated eye. At fourth-year, of 54 graduated, 59 % felt comfortable in evaluate optic disc, 46 %, macula, 61 %, vessels and 57 %, retina; 11 % felt very confident in fundoscopy, 65 % somewhat confident, and 26 % not confident. In our study, this decrease in self-confidence was observed in PO group, but not in CO group, which will be discussed next.

### Comparison of both devices in diagnostic accuracy

A few studies have already compared CO and PO for accuracy in diabetic retinopathy screening [3], cup-to-disc ratio evaluation [21] and optic nerve pathologies at emergency room [2]. The first study to compare both devices enrolled 8 first-year MS for cup-to-disc ratio evaluation and ease of use. Student were assigned to use one ophthalmoscope for patient examination in one session and then swap the device for a second session^21^. No difference was found in cup-to-disc ratio measurement between CO and PO (0.08 ± 0.14 vs. 0.09 ± 0.13, *p* = 0.67) in the first session. However, there was a significant difference in the second session regarding cup-to-disc ratio (p = 0.02), which suggests a learning effect. Moreover, MS scored PO easier to use than CO (9 vs. 8, *p* < 0.0001), and dilated easier than undilated for each device, despite no difference was found when compared dilated CO examination versus undilated PO (*p* = 0.82)^21^. Another comparison was made for diagnose of optic nerve pathologies in emergency environment by Petrushkin et al. [2], which included 36 emergency doctors who examined critical patients with both devices. PO had a higher sensitivity and specificity (0.63 and 0.55) than CO (0.31 and 0.30), which was statistically significant (*p* = 0.03 in both comparisons); also, doctors (*p* = 0.001) and patients (*P* = 0.04) preferred PO over CO. In terms of diabetic retinopathy, Tan et al. [3] evaluated sensitivity and specificity of PO and CO to diagnose sight-threatening diabetic retinopathy using indirect ophthalmoscopy at slit-lamp biomicroscopy as reference. To avoid intra- and inter-rater variability, only one examiner performed all the evaluations. In a total of 200 patients, CO had a higher sensitivity (73.2 % vs. 58.5 %), equal specificity (93.7 %), higher positive predictive value (75 % vs. 70.6 %) and higher negative predictive value (93.1 % vs. 89.8 %) compared to PO, respectively. Also, CO was considered 1.38 times easy-to use than PO.

### Direct ophthalmoscopes versus smartphone-based ophthalmoscopes

Beyond the comparison of CO and PO (direct ophthalmoscopes), Dunn et al [29] compared also these two devices to a non-mydriatic camera and three SF (PO + iExaminer, an smartphone adaptor and D-EYE), using a questionnaire that ranged from 6 (lowest score) to 30 (highest score). One hundred and forty-six medical students had their perceived usefulness, ease of use, ease of view, confidence and quality of training measured with each of the 6 devices after a 10-minute practical training. SF had the highest score for perceived usefulness (24.81) when compared to non-mydriatic camera (22.45, *p* < 0.001) and direct ophthalmoscopes (22.95, *p* = 0.006). For easier of use, SF also had a higher score (25.37) when compared to the previous 2 categories (21.55, *p* < 0.001) and 22.96, *p* < 0.001). Regarding ease to view CO showed an inferior score when compared to the other 5 devices (*p* < 0.007); while confidence with CO only had difference with PO (*p* = 0.001), which this second obtained the highest confidence level, and no difference with the other devices. Quality of training had no difference among the 6 devices. Kim et al [30] also compared CO to D-EYE in terms of visualization of optic nerve and blood vessels, along with confidence, by second year MS after 1-h practical training. Among 101 students enrolled, 82.3 % reported visualization of the optic nerve in an undilated pupil with the SF, while 48.5 % reported the same with the traditional device (*p* < 0.0001). MS also performed better with the first device even with dilated pupils (85.9 % vs. 65.4 %, *p* = 0.0036). Ease of use (4.25 vs. 2.76, *p* < 0.0001) and confidence (3.70 vs. 2.41, *p* < 0.0001) were also higher for D-EYE. Moreover, MS preferred the new device than the traditional one (78.2 % vs. 77.2 %, *p* < 0.0001). However, a study that compared CO to a 20 diopters lens plus smartphone camera [31] in identify the optic nerve head, macula and retina vessels found a different outcome. Among 137 MS enrolled for the 20 to 25-minute instruction in one device or another, they referred a higher quality of visualization of optic nerve head and macula in CO than in the SF, with less attempts need to identify the structures with CO (2.7 ± 2.3 vs. 4.5 ± 2.9, *p* < 0.001). CO was preferred over the SF for 69 % of MS, and 24 % preferred this over CO. These studies have revealed that smartphone-based fundoscopy is an emerging technique for fundus examination teaching, but more investigation is needed before it become the choice method of teaching fundoscopy due to a variety of different SF devices and the useful of other non-traditional direct devices like wide field ophthalmoscopes.

Not restricted to direct or SF ophthalmoscopes, another useful technique of fundus examination is the non-mydriatic fundus photograph, which was evaluated in the emergency environment by Bruce et al [32] and Dunn et al [33] in terms of ophthalmoscopy skills and diagnostic accuracy. The first study [32] compared direct ophthalmoscope to non-mydriatic fundus photograph in an emergency environment. Among the 354 patients included, 239 fundus photographs (68 %) were reviewed: 125 of these (35 %) were considered helpful in the evaluation, 16 of 35 (46 %) relevant findings were identified. The sensitivity of fundus photographs in identify neurological emergency pathologies was 46 % and specificity 91 %. The second study [33] enrolled 345 patients for fundus photograph, which 283 (45 %) were considered the method useful. The sensitivity for emergency physicians detect urgent conditions with fundus photograph evaluation was 40 % and specificity was 82 %. These studies shows that, even with different devices, fundus evaluation is still a major point of concern on medical education due to a limited proficiency in the exam, which reflects in low diagnostic accuracy.

### Factors that influence long-term self-confidence

Our study provides the longest follow-up comparison between CO an PO in terms of evaluate the main fundus structures after a practical training. Lee et al. [34], in a relatively similar study design, assessed self-confidence of second year MS with a 6-point Likert scale questionnaire, divided in groups of 8–9 students, pre- and post- a single 90-min session practical training in both PO and CO. Of the session, only 40 min were dedicated to teach direct ophthalmoscopy. Of the 172 students that completed the pre-session survey and 108 that completed the post-session survey, an increase of the confidence in visualize the optic nerve was reported with the PO (1.21 vs. 4.48, *p *< 0.001). Optic nerve was observed by 99.1 % of MS with either devices. However, confidence with CO, which was higher than PO before training (2.30 vs. 1.16, *p* < 0.001), showed a worst performance after training (3.93 vs. 4.49, *p* < 0.001). Moreover, 80 of 85 students reported preference for the PO device. Eight months after the training, 42 students were re-evaluated in their ophthalmoscopy skills. They performed fundoscopy in two patients. 57.1 % of MS responded the evaluation correctly, and 40.5 % responded incorrectly with PO, while 9.5 % responded correctly and 23.8 % responded incorrectly with CO. Moreover, 97.6 % and 33.3 % visualized the optic nerve with the respective PO and CO devices.

In our casuistic, a superiority of PO was expected due to its larger field of view and magnification, but CO superiority 1 year after intervention was not expected as demonstrated by the studies previously described. Some hypothesis can be listed to explain these findings: (1) lower cost of conventional ophthalmoscopes models compared to the PanOptic, which facilitates ownership of the first one, (2) availability of conventional ophthalmoscopes in some sectors of our hospital and primary-care units, which propitiated MS contact with CO during the period but not with PO, (3) and the longest training session, when compared to other studies, would provide a better retention with CO than with PO. Some aspects that influence the confidence in fundoscopy were investigate by Schulz et al [20], which compared MS of the 4th -year to MS of final year of medicine course. The authors found that the first group was more confident in perform the examination than the second group, and described the exposure to an abnormal fundus and the feedback on their ability in using the ophthalmoscope as the factors that influenced this outcome. Also, the authors suggested an early formal instruction, with a reinforcement in the final year, as an attitude to improved MS confidence in fundoscopy. Moreover, McNaught and colleagues [13] related the ownership of ophthalmoscopes by MS with the frequency that fundus examination was performed: among those who owned a device, 7.3 % always performed fundoscopy, while 26.8 % did half of the time and 66 % occasionally. Among the group that did not owned an ophthalmoscope, only 4.3 % always performed the exam, 17.4 % did half of the time and 78.3 % occasionally. These differences, however, were not statistically significant, which reflects that ownership of ophthalmoscope may not be a determinant factor for fundoscopy practice.

### Strengths

Our study translated and validated to Portuguese a questionnaire that assessed the self-confidence of medical students in evaluate the most critical structures in eye fundus. This will provide a standardized methodology for future studies that aim to evaluate the same subject and allow comparison of the results.

Compared to literature, our training session is the longest to the best of our knowledge (6 h). In addition, our study provides the longest comparison of self-confidence of MS to exam eye fundus (1 year) with two different devices. This long term analysis allowed us to discuss factors that influenced the self-confidence with two different devices during the 1-year period, which will provide a basis for future interventions that aim to increase the confidence in MS handle an ophthalmoscope.

### Limitations

One main issue about cross-cultural validation is the meaning of “equivalence” [35]: grammatical equivalence and automatic translation does not mean semantic equivalence necessarily, and adaptations must be made in order to keep it relevant and representative of the new culture; a failure in this item would generate an instrument that does not measure the same parameters of the original one. Furthermore, availability of native English-speakers for the back-translation sometimes is limited in some locations [26].

Because of Covid-19 outbreak and consequent social distancing, our results should be interpreted with certain caution. Several activities in our institution were suspended for a course of 3 months, with all the focus kept on emergency assistance, where clerks did not act during this period. Moreover, almost after 1 year of the pandemic, most practical activities were not fully resumed, which certainly impacted the total of patients’ fundus examined by MS during the last year.

We were not able to measure students’ self-confidence before practical training, and compared both intervention groups to a different control group, which limited a before-after analysis of the intervention. Also, we did not evaluate students’ skills in fundus examination, that would provide additional information, given that self-confidence not always correlate with performance. At 1-year questionnaire re-application, PO group had a lower rate of answers, in opposition to the CO group, what could had constituted a bias. Ownership of ophthalmoscopes, which would help a more accurate explanation of our results, was not evaluated also.

## Conclusions

In conclusion, medical students were more confident in use PO as an instrument to perform direct ophthalmoscopy immediately after practical training, but confidence level of CO was higher compared to PO 1 year after practical ophthalmoscopy training.

We suggest more studies with wide-field ophthalmoscope to better evaluate advantages and disadvantages of these devices in long-term on self-confidence, technique performance and diagnostic accuracy. Comparison with other emerging techniques of fundoscopy, like smartphone-based, would also provide additional information for the decision of the choice method to teach fundus examination to MS in medical schools.

## Supplementary Information

**Additional file 1.**

## Data Availability

The datasets used and/or analyzed during the current study are available from the corresponding author on reasonable request. All data generated or analyzed during this study are included in this published article and its supplementary information files.

## Abbreviations

MS: Medical students
SF: Smartphone-based ophthalmoscope
CO: Conventional ophthalmoscope group
PO: PanOptic ophthalmoscope group
