# Supplementary material for: Comparison of conventional and wide field direct ophthalmoscopy on medical students’ self-confidence for fundus examination: a 1-year follow-up
Source: BMC Med Educ. 2021 Sep 26;21:507. doi: 10.1186/s12909-021-02942-y (PMC8474948; doi:10.1186/s12909-021-02942-y)
Supplement: Supplementary file 1 — Additional file 1. [file 12909_2021_2942_MOESM1_ESM.docx]

**Nível de confiança global e no exame do disco e da mácula**

**Iniciais ______________**

**Nível de confiança (circule apenas uma)**

|  | Nenhum | Pouco | Regular | Bom | Ótimo |
| --- | --- | --- | --- | --- | --- |
| Em geral, com a técnica de fundoscopia | 1 | 2 | 3 | 4 | 5 |
| Avaliar a relação escavação-disco | 1 | 2 | 3 | 4 | 5 |
| Avaliar as margens do disco | 1 | 2 | 3 | 4 | 5 |
| Diferenciar mácula saudável versus mácula patológica | 1 | 2 | 3 | 4 | 5 |
